# Needle Cricothyroidotomy by Intensive Care Paramedics

**DOI:** 10.1017/S1049023X22001157

**Published:** 2022-10

**Authors:** Rembrandt Bye, Toby St Clair, Ashleigh Delorenzo, Kelly-Ann Bowles, Karen Smith

**Affiliations:** 1. Department of Paramedicine, Monash University, Victoria, Australia; 2. Ambulance Victoria, Victoria, Australia; 3. The Royal Children’s Hospital, Department of Trauma, Melbourne, Australia

**Keywords:** advanced airway management, cricothyroidotomy, prehospital

## Abstract

**Objective:**

Cricothyroidotomy is an advanced airway procedure for critically ill or injured patients. In Victoria, Australia, intensive care paramedics (ICPs) perform needle cricothyroidotomy utilizing the proprietary QuickTrach II (QTII) device. Recently, an Ambulance Victoria (AV) institutional change in workflow included pre-puncture surgical incision to assist in successful placement. This review aims to explore whether a surgical pre-incision prior to the insertion of the device improved overall procedural success rates of needle cricothyroidotomy using the QTII.

**Methods:**

This was a retrospective review of all patients who received a needle cricothyroidotomy by ICPs from May 1, 2015 through September 15, 2020. Data and patient care records were sourced from the AV data warehouse.

**Results:**

A total of 27 patients underwent a needle cricothyroidotomy with the mean age of patients being 50.2 years. Most cricothyroidotomies were performed using the QuickTrach II kit (92.6%). Prior to modification of the QTII procedure, front-of-neck access (FONA) success was 50.0%; however, this improved to 82.4% after the procedures recent update. The overall success rate of all paramedic-performed needle cricothyroidotomy during the study period was 74.1% (n = 20).

**Conclusions:**

This review demonstrates that propriety devices such as the QTII device achieve a low success rate for a FONA intervention. Despite the low frequency of this procedure, ICPs with extensive training and regular maintenance can perform needle cricothyroidotomy using scalpel assistance with a reasonable success rate. But when compared to the broader literature, success rate using a more straightforward technique such as a surgical cricothyroidotomy technique is likely going to be higher.

## Introduction

Establishing a definitive airway is a priority when managing critically ill or injured patients in the prehospital environment, enabling targeted ventilation and oxygenation, as well as protection against aspiration.^1,2^ There are a broad range of maneuvers, adjuncts, and interventions that can assist with maintaining airway patency, including continuous bag-valve-mask ventilation, supraglottic airway devices, and endotracheal tube placement. In circumstances where these measures fail leading to a “cannot ventilate, cannot oxygen” situation, a rescue cricothyroidotomy may be required.

Cricothyroidotomy, a rapidly performed form of front-of-neck access (FONA) technique, enables critical oxygenation and ventilation by the way of percutaneous airway access. This is performed by either a needle or surgical approach. The preferred method of cricothyroidotomy remains unknown, despite a prehospital meta-analysis published in 2010 which identified no statistically greater technique.^1^ However, numerous studies have since been published which may provide another opportunity for a follow-up assessment of the most effective technique.^2–4^ Although a superior technique is yet to be statistically determined, surgical cricothyroidotomy techniques appear to be a more commonly adopted technique for FONA.^5^

There is a paucity of literature that examines the success of needle cricothyroidotomy devices within the prehospital environment. In Victoria, Australia, intensive care paramedics (ICPs) performed FONA using a needle cricothyroidotomy technique. This review has sought to explore the incidence, indications, and success rates of cricothyroidotomy performed by ICPs.

## Methods

### Study Design

This was a retrospective review of all patients who underwent a cricothyroidotomy by ICPs from May 1, 2015 through September 15, 2020. Patients of all ages were included.

### Setting

Ambulance Victoria (AV) is the sole provider of Emergency Medical Services for Victoria, Australia, providing care to more than 6.5 million people over 220,000 square kilometres.^6^ This system is a two-tiered emergency response which includes Advanced Life Support (ALS) and ICPs. Over 330,500 emergency patients in 2018-2019 were treated by AV paramedics.^7^ Only ICPs are endorsed to perform cricothyroidotomy. To gain authority to this level of practice, an ALS paramedic must complete a three-year undergraduate degree followed by a 12-month supervised graduate program. Following two years of clinical experience, an ALS paramedic is eligible to begin training to become an ICP. To operate as an ICP, these paramedics are required to complete a further 12 months of postgraduate study in addition to a supervised 12-month, on-road training to become a qualified ICP.

The clinical skill set of ICPs includes but is not limited to advanced analgesia, intraosseous insertion, thrombolysis, anti-arrhythmic pharmacology, rapid sequence induction (RSI), intubation, and cricothyroidotomy.

### Procedure

Cricothyroidotomy is performed in accordance to AV’s clinical work (procedural) instructions.^8^ Currently, ICPs perform needle cricothyroidotomy using the commercial QuickTrach II (QTII; VBM Medizintechnik GmbH; Neckar, Germany) device.^9^ The QTII device is made up of a solid trocar needle cannula with a preloaded 4.0mm internal diameter cuffed catheter. The device is inserted through the cricothyroid membrane and into the tracheal lumen where the needle is removed, and a cuffed ventilation catheter is then left in place creating a definitive small lumen airway.

In this setting, authorized indications for cricothyroidotomy by Victorian ICPs are mentioned in Table 1. The QTII is a commercially produced cuffed airway device and was first introduced in May 2015 to replace the uncuffed Portex Mint-Trach kit (Smith’s Medical; Minneapolis, Minnesota USA). The initial insertion of QTII is confirmed via the aspiration of a syringe plunger attached to the trocar device, but overall success is confirmed by continuous tracing of electronic end-tidal waveform capnography (EtCO2). In August 2017, following reports of device-related complications from operational ICPs, the clinical work instructions adjusted the protocol to include a small surgical incision into the skin prior to insertion of the QTII device. This was to be performed over the cricoid membrane landmark and was aimed at improving the success of insertion of the QTII device. A scalpel incision prior to insertion of the QTII device was officially implemented into clinical practice from September 2017. Despite performing a surgical incision to expose the cricoid membrane for the insertion of the QTII device, the authors still define this procedure and its mechanism as a puncture-based needle cricothyroidotomy technique as described in the literature.^5^

**Table 1.** Ambulance Victoria Indications for Cricothyroidotomy

RSI has been attempted but intubation was not achieved Massive facial trauma is present, and RSI is deemed unsafe RSI not possible due to lack of IV/IO access Upper airway obstruction due to pharyngeal oedema or highly impacted foreign body which is unable be dislodged from manual techniques or Magill’s forceps

Abbreviations: RSA, rapid sequence induction; IV, intravenous; IO, intraosseous.

Prior to the introduction of the QTII device, FONA was performed using the Portex Mini Trach kit. However, due to the operational challenges that can occur when a new procedure is being introduced into clinical practice, the Mini-Trach device was still available to some parts of the operational ICP workforce, and as a result, several of the early cases of cricothyroidotomy that occurred at the start of the study period involved the ICP performing a needle cricothyroidotomy using the Mini-Trach device.

### Data Source

Data were sourced from the AV data warehouse. Operational paramedics record the details of all emergency case attendances in an electronic patient care record, which is then stored in the AV data warehouse. For the present study, the warehouse was searched for all cases where cricothyroidotomy was performed by ICPs from May 1, 2015 through September 15, 2020. The required data were provided to the project investigators using a password protected Microsoft Excel spreadsheet (Microsoft Corp.; Redmond, Washington USA).

### Statistical Analysis

Using Microsoft Excel, descriptive statistics were obtained on patient’s demographics, etiology, indications of use, and technique along with overall success rates (Table 2 and Table 3). Categorical data include frequencies and proportions and all available continuous variables have been described using means and standard deviation (SD).

**Table 2.** Sub-Group Analysis of Needle Cricothyroidotomy Cases

	No. of Patients (%)	No. Successful (%)
**Trauma**		
Motor Vehicle Crash	8 (61.5)	5 (62.5)
Thermal Injury	2 (15.4)	2 (100)
Motorcycle Crash	1 (7.7)	0 (0)
Assault	1 (7.7)	1 (100)
Gunshot	1 (7.7)	1 (100)
**Medical**		
Cardiac Arrest	7 (50.0)	7 (100)
Airway Obstruction	4 (28.6)	4 (100)
Anaphylaxis	3 (21.4)	0 (0)
**Indication**		
Failed Intubation	17 (63.0)	14 (82.4)
Primary	10 (37.0)	6 (60.0)
**Technique**		
Mini Trach	2 (7.4)	2 (100)
QuickTrach II without Pre-Incision	8 (29.6)	4 (50.0)
QuickTrach II with Pre-Incision	17 (63.0)	14 (82.4)
**ICP Cohort**		
Road	18 (66.7)	14 (82.4)
Flight	9 (33.3)	6 (66.7)

Abbreviation: ICP, intensive care paramedic.

**Table 3.** Summary of Recent Cricothyroidotomy Studies

	Peters^10^	Kyle^11^	Bell^4^	High^12^	Schober^13^
Publication Date	2015	2016	2017	2018	2019
Provider	RN	CM	CP	RN, CP	P
Total Performed (No.)	30	86	36	13	18
Technique	S	S	S	S, N	S, N
Success Rate (%)	96.7%	92.0%	97.0%	100%	94.0%

Abbreviations: RN, flight nurse; CP, critical paramedic; P, physician; CM, combat medic; S, surgical cricothyroidotomy; N, needle cricothyroidotomy.

### Ethics

Ethical approval (approval no. 25291) for this retrospective review was granted by the Monash University Research Ethics Committee (Victoria, Australia).

## Results

**Figure 1.**
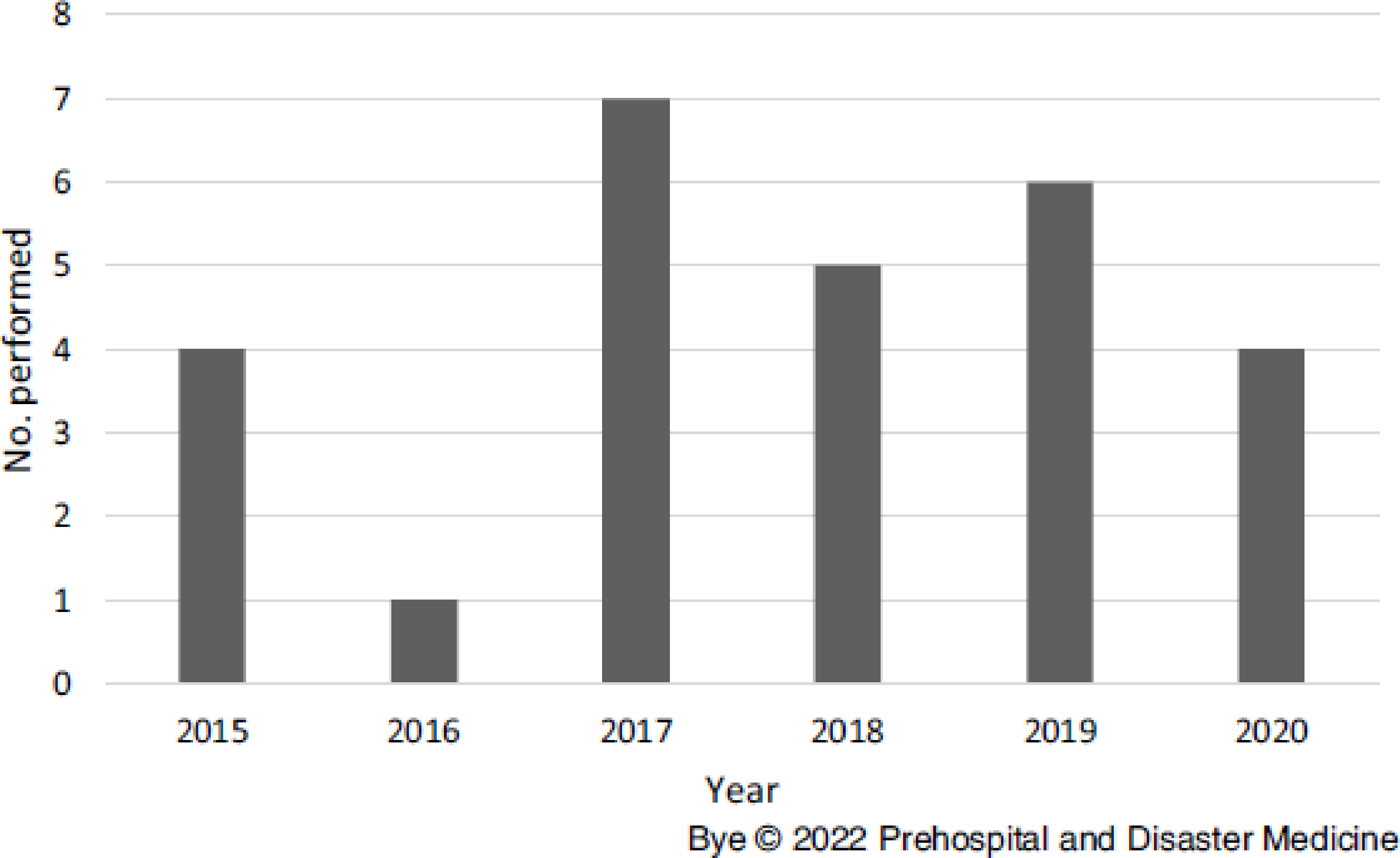
Distribution of Cricothyroidotomies per Year.

The database search originally yielded 29 unique cases; however, two cases were excluded (Figure 1). One due to a patient receiving QTII device placement during a pre-existing tracheostomy emergency, and the second due to a cricothyroidotomy performed by a neighboring state service in which the patient was transported by AV ICPs. No children under the age of 18 years received a cricothyroidotomy during the study period.

The mean age of patients who received needle cricothyroidotomy was 50.2 years (SD = 18.5) and most patients were male (n = 23; 85.2%). Prior to the procedure, the mean Glasgow Coma Score (GCS) was four and up to 81.5% of patients that received a needle cricothyroidotomy had a GCS of three.

There were 14 (51.9%) cricothyroidotomy performed as a result of medical causes and 13 (48.1%) performed due to trauma cases. A sub-analysis of the specific etiologies for cricothyroidotomy use was determined from manual examination of patient care records, as shown in Table 2.

Overall, the majority of cricothyroidotomies were performed by road-based ICPs (66.7%) with the remaining performed by the aeromedical ICPs (Table 2). All cricothyroidotomy indications were categorized as either being a part of the failed intubation algorithm or performed as the primary airway management intervention (Table 3 ^4^,^10–13^).

The overall success rate for paramedic-performed needle cricothyroidotomy was 74.1% (n = 20). Success was confirmed with electronic waveform capnography, however numerical EtCO2 data could only be obtained for 18 cases. The mean EtCO2 was 37.9 (SD = 12.7). Numerical EtCO2 data were not available for two successful cricothyroidotomy cases. However, upon manual review of patient care records, confirmation of success was confirmed using an EtCO2 colorimetric device.

The introduction of the QTII procedural modification in 2017 increased success rates from 50.0% to 82.4%. Initially, a Fisher’s exact P value of .365 suggested no significance between the two techniques. However, due to the small total sample size, any generated P value may not necessarily reflect the true clinical beneficence between the two QTII techniques. Due to the overall sample size, a logistic regression was then performed and identified that the QTII placement with scalpel assistance had non-significant trend towards greater success rate compared the QTII without surgical scalpel incision (OR: 3.11; [0.53, 18.38]; P = .210).

## Discussion

Establishing a definitive airway is vital for critically ill or injured patients to provide life-saving oxygenation and ventilation. Securing a definitive airway by performing a cricothyroidotomy is often described as an infrequently performed, high-risk procedure.^14^ In this study, the characteristics and success rates are described of different needle techniques in patients undergoing cricothyroidotomy by ICPs in Victoria. An overall placement success rate of 74.1% was observed. Following the introduction of a pre-puncture incision followed by placement of the QTII device, success rate improved from 50.0% to 82.4%.

Few studies describe paramedic-performed cricothyroidotomy, however the overall success rate in this study differs from a recent paramedic study in the United Kingdom. Bell reported advanced care paramedics achieved a cricothyroidotomy success rate of 97%.^4^ This is higher than the success reported in this study, however Bell utilized the surgical technique like other recently published studies, which may account for the different success rate (Table 3). Following the introduction of the pre-placement surgical incision, the reported success rate increased to 82.4%. This may suggest that a FONA device with scalpel assistance or surgical cricothyroidotomy technique may be more superior to the sole needle cricothyroidotomy technique. This is yet to be reflected in the literature due to the limited sample sizes of this infrequent procedure. As a result of the QTII with a scalpel incision being performed most often during this study period, this success rate of 82.4% is more appropriately comparable against the Bell paramedic study.^4^ However, due to the incomparable differences regarding education, training requirements, airway management protocol, availability of paramedic-performed drug facilitated intubation, and the exposure to high-acuity cases, the results from that study are not generalizable and may not reflect other studies (Table 3). Further research is required to compare the different airway management protocols and decision-making factors between the different jurisdictions regarding cricothyroidotomy.

The recommended instructional use of the QTII device specifies that there is no requirement for a surgical incision over the cricoid membrane landmark site before percutaneous insertion, due to the sharp and tapered trocar needle.^9^ However, from this retrospective review, the manufacturing recommended procedure may require review with the option to perform a scalpel incision prior to insertion, as two of the failed procedures were due to the QTII trocar needle not being long enough to be confidently secured in the tracheal lumen airway (ie, obesity and enlarged neck). For example, the incidence of a difficult intubation and FONA is more likely to occur in obese patients when compared to non-obese patients.^15^ However, no evidence currently can support that the QTII device failure is due to difficult airway anatomy.

Prior to the introduction of surgical scalpel assistance for the QTII, the recommended instructional use of this device was in clinical practice for 27 months. Only eight cricothyroidotomies were performed using the device’s recommended instruction of use, which ultimately achieved an overall success rate of 50.0% (n = 4). In comparison, the updated scalpel incision method was in clinical practice for 37 months. This is the first study to the authors’ knowledge that describes the use of a surgical scalpel prior to inserting the QTII device.

Across the five-year study period, a failed intubation (n = 17; 63.0%) was the most common indication for needle cricothyroidotomy compared to primary airway intervention (n = 10; 37.0%). Among the 17 intubations, Cormack-Lehane (CL) airway grade classification data were available in 16 cases. Grade 4 CL airway occurred in 11 cases (68.8%) of the failed intubation indications for cricothyroidotomy. Airway grade has been demonstrated to influence success rates of intubation.^11^ The requirement for FONA interventions may be more predominant for Grade 3 or Grade 4 CL airways due to factors such as laryngeal injury, oedema, inflammation, old age, obesity, and manual inline stabilization in trauma patient where the ability of gaining the most optimal airway position is limited. These factors can affect the ability to achieve first-pass or overall endotracheal intubation success rates.^16,17^ It is essential that clinicians can promptly identify a difficult airway that may require FONA and decide when to escalate to cricothyroidotomy. Of the 17 performed intubations prior to needle cricothyroidotomy, only eight of the failed intubation attempts were following neuro-muscular blocking agents in RSI with the remaining incidents in the setting of unassisted intubation, such as cardiac arrest. During this period, a total of 4,617 ICP RSI procedures were performed by AV ICPs. The incidence of RSI failed cricothyroidotomies was 1.7 per 1,000 RSI. Seven of the eight failed RSI indicated cricothyroidotomies were successful, confirmed by electronic waveform capnography.

Performing a cricothyroidotomy is a high-risk procedure where complications such as the inability to advance through the skin, pierced epiglottitis, and hemorrhage may occur.^18,19^ In this study, three complications were reported when needle cricothyroidotomy was performed using the QTII device. Two of these complications occurred in the seven failed needle cricothyroidotomies. Neck subcutaneous emphysema was most the common (n = 2) complication to occur, but when examining previous cricothyroidotomy studies, no incidences of neck subcutaneous emphysema could be identified.^4,10,13,20^ In addition, no evidence exists that reflects if the incidence of neck subcutaneous emphysema only occurs from a percutaneous device such as the QTII.^5^ The final complication reported was that the initial surgical scalpel incision site was performed too high, however this was corrected and the needle cricothyroidotomy was inserted successfully.

Cardiopulmonary resuscitation (CPR) was simultaneously performed in 55.6% (n = 15) of cases. For two cases, the cricothyroidotomy performing ICPs requested CPR to be temporarily ceased to enable procedure safety, and for the remaining 13 cases, CPR was actively performed. Needle cricothyroidotomy along with simultaneous CPR was successfully performed in 12 cases (92.3%). No long-term patient outcomes were followed up with receiving hospitals, however based on the data obtained, resuscitation was ceased on-scene 40.7% (n = 11) of the time prior to transport to hospital.

Emergency cricothyroidotomy is often performed in the “cannot intubate, cannot ventilate” situation, and as such, identification of the optimal technique is an important factor when designing a prehospital difficult airway algorithm.

## Limitations

Since the current study was a retrospective review, data accuracy and recording of variables relied upon the judgement and incident recall by the attending ICP. Victorian ICPs in this study have undergone extensive training and are often exposed to high-acuity patients. The results from this study may not be transferable to other prehospital care providers. Lastly, comprehensive statistical analysis comparing the multiple sub-analysis groups (Table 2) were not performed due to the small sample sizes.

## Conclusion

This review adds to the limited literature surrounding paramedic-performed FONA procedures and may guide other prehospital services regarding their approach to developing FONA guidelines. Due this procedure’s rarity, performing a future prospective study that compares this study’s needle cricothyroidotomy technique with a potentially more beneficial technique within the prehospital environment would be challenging. But based on the initial period of this review’s timeline, needle cricothyroidotomy within the prehospital environment is likely to obtain an unacceptable success rate for critically ill and injured patients; when compared to the literature, higher overall success rates were more common when using a surgical cricothyroidotomy technique. However, based on this review alone, success rates did improve when the placement of the QTII cricothyroidotomy device occurred when utilized with a surgical scalpel incision prior to insertion.
